# Tackling equitable coverage and quality of care for neonates in hospitals: a pre-post assessment on asphyxia interventions in Mesoamerica

**DOI:** 10.1186/s12887-021-02999-0

**Published:** 2021-12-01

**Authors:** Aruna M. Kamath, Maximilian G. Thom, Casey K. Johanns, Katie Panhorst Harris, Karla Schwarzbauer, José C. Ochoa, Paola Zuniga-Brenes, Diego Rios-Zertuche, Ali H. Mokdad, Bernardo Hernandez

**Affiliations:** 1 Institute for Health Metrics and Evaluation, University of Washington, 3980 15th Ave NE, Seattle, WA 98195 USA; 2 Department of Anesthesiology, University of Washington, Seattle, WA USA; 3 Inter-American Development Bank, Washington, DC USA; 4 Inter-American Development Bank, San José, Costa Rica

**Keywords:** Birth asphyxia, Intra-partum related hypoxia, Neonate, Newborn, Quality of care, Central America, Mexico, Honduras, Nicaragua, Guatemala

## Abstract

**Background:**

Intrapartum-related hypoxic events, or birth asphyxia, causes one-fourth of neonatal deaths globally and in Mesoamerica. Multidimensional care for asphyxia must be implemented to ensure timely and effective care of newborns. Salud Mesoamérica Initiative (SMI) is a performance-based program seeking to improve maternal and child health for low-income areas of Central America. Our objective was to assess the impact of SMI on neonatal asphyxia care in health centers and hospitals in the region.

**Methods:**

A pre-post design. Two hundred forty-eight cases of asphyxia were randomly selected from medical records at baseline (2011–2013) and at second-phase follow-up (2017–2018) in Mexico (state of Chiapas), Honduras, Nicaragua, and Guatemala as part of the SMI Initiative evaluation. A facility survey was conducted to assess quality of health care and the management of asphyxia. The primary outcome was coverage of multidimensional care for the management of asphyxia, consisting of a skilled provider presence at birth, immediate assessment, initial stabilization, and appropriate resuscitation measures of the newborn. Data were analyzed using multivariable logistic regression.

**Results:**

Management of asphyxia improved significantly after SMI. Proper care of asphyxia in intervention areas was better (OR = 2.4; 95% CI = 1.3–4.6) compared to baseline. Additionally, multidimensional care was significantly higher in Honduras (OR = 4.0; 95% CI = 1.4–12.0) than in Mexico. Of the four multidimensional care components, resuscitation showed the greatest progress by follow-up (65.7%) compared to baseline (38.7%).

**Conclusion:**

SMI improved the care for neonatal asphyxia management across all levels of health care in all countries. Our findings show that proper training and adequate supplies can improve health outcomes in low-income communities. SMI provides a model for improving health care in other settings.

## Background

Intrapartum-related hypoxic events – commonly referred to as birth asphyxia and defined as “failure to initiate and sustain breathing at birth” [1] – causes approximately one-fourth of neonatal deaths globally [2]. For the Mesoamerican countries of Honduras, Guatemala, Nicaragua, and Mexico, birth asphyxia remains a leading cause of neonatal deaths, with minimal progress prior to this study from 1990 to 2010, as reported by the Global Burden of Disease study [3, 4]. Life-saving interventions to detect and manage asphyxia during the first minute of life are best suited for hospital settings, with a reduction potential of 30% [5]. Revised by WHO, UNICEF, and UNFPA in 2009, the Emergency Obstetric and Newborn Care (EmONC) guidelines [6] focus on quality of care for labor and delivery. The EmONC services call for nine essential signal functions, of which basic newborn resuscitation is a required function regardless of facility type.

To address this performance gap in hospitals, combined rather than single interventions must be implemented, monitored, and evaluated to ensure effective and timely care for newborns. The Salud Mesoamérica Initiative (SMI) is an innovative, performance-based program seeking to improve health for the most impoverished 1.8 million women and children on a regional level, from southern Mexico through Central America [7, 8]. The ministries of health set forth country-led performance indicators and associated incentivized target goals that were monitored by SMI [9]. These performance indicators, or interventions, sought to integrate maternal and child services across the health system, rather than in silos. As such, asphyxia care interventions were measured alongside maternal targets during labor and delivery, as well as antenatal, postpartum, and older child health goals [10].

The primary outcome of this analysis is multidimensional care coverage for newborn asphyxia in health facilities, consisting of four components. First, a skilled birth provider presence for all newborn deliveries is expected. Second, immediate assessment with Apgar scoring to detect newborn distress is recorded. Third, initial stabilization with drying, heat application, and, if needed, stimulation to breathe is monitored. Fourth, for newborns requiring additional assistance, provision of basic resuscitation with bag-mask ventilation, or advanced resuscitation such as the use of chest compressions and endotracheal intubation is assessed. As such, the objective of this study is to evaluate clinical performance of asphyxia management in low-resource Mesoamerican hospitals by examining multidimensional care coverage and quality of care before and after implementation of SMI.

## Methods

### Design

This present analysis on neonatal asphyxia utilizes a subset of data collected for the evaluation of SMI, a multinational maternal and child health initiative based in low-income sectors of Mesoamerica. Using a pre-post study design, data collection occurred at the baseline (March 1, 2011, to August 31, 2013) and second-phase follow-up (June 1, 2017, to August 30, 2018) after quality-of-care interventions were implemented. Under SMI, health system strengthening was performed in a stepwise process from 2012 to 2018. For asphyxia care, the first phase of SMI focused on availability of inputs, such as staffing skilled providers and having the medications and equipment necessary for neonatal resuscitation. Highlighted in this study, the second phase of SMI sought to increase quality and coverage of services. Interventions focused on training within broader quality improvement strategies.

Qualified personnel were trained in neonatal resuscitation concentrating on the four multidimensional asphyxia care components measured in this analysis, alongside general newborn care such as temperature control and umbilical cord care, within 3 years prior to second-phase follow-up. Quality improvement strategies sought to translate simulation-based training into real-life settings, with monitoring and evaluating using a Plan-Do-Study-Act cycle. Quality improvement steps involved creating flowcharts to map steps needed to provide care and designate personnel roles, standardizing work processes, measuring performance of the four multidimensional components of asphyxia care, and investigating root causes of underperformance. Technical assistance was provided by SMI to hospitals, with coaching and support throughout quality improvement cycles. Neonatal interventions were customized to the needs of each hospital. Health facilities measured the four multidimensional care components in this analysis in order to timely diagnose and provide asphyxia care at bedside. In most settings, interventions also included updating national protocols based on the latest evidence-based asphyxia practices, certification of general physicians and nurses to manage neonatal asphyxia to address shortages in availability of pediatricians and neonatologists, policy to preemptively request personnel to manage asphyxia for high-risk deliveries, and availability of pre-made asphyxia kits in delivery rooms.

### Population and sample

Fifty-eight health facilities at baseline and 71 health facilities at second follow-up were visited in Mexico (health jurisdictions of Ocosingo, Palenque, Pichucalco, and San Cristobal De Las Casas); Nicaragua (departments of Bilwi, Jinotega, Las Minas, Matagalpa, and North Atlantic Region); Honduras (departments of Choluteca, Copan, Intibuca, La Paz, Lempira, Octoepeque, and Olancho); and Guatemala (departments of Huehuetenango and San Marcos) participated in this study. Due to the limited number of facilities, all facilities serving the lowest income (one-fifth of the population) per census data that met the EmONC criteria of basic-level facilities (health centers with basic neonatal resuscitation with bag-mask ventilation and routine maternal delivery care) or comprehensive-level facilities (referral hospitals with surgical and transfusion capabilities) were included. Ambulatory-level facilities (without neonatal asphyxia care capability), as well as care started elsewhere (transferred care) were excluded.

In terms of the medical record sample selection, records were first identified by the International Classification of Disease coding (ICD-10) of newborn asphyxia diagnosis and other neonatal conditions at the time of delivery. Due to this collection method, deliveries with healthy outcomes fell under a separate survey module and were not captured for this analysis. Next, records at each facility were sampled at random using a systematic sampling method, with the sampling interval corresponding to the sampling fraction. A random starting point was chosen, marking a two-year timeframe. Sampling quotas varied, impacted by facility and country resources. Sample sizes had enough power to detect differences in evaluation indicators, such as treatment of neonatal complications according to national norms.

### Procedure

During data collection rounds, a health facility survey was performed, consisting of an interview questionnaire to assess facility workforce and infrastructure; an observation checklist to document available medical equipment and drug stock; and a medical record review to determine neonatal care coverage. Hired as independent surveyors, local physicians and nurses were instructed on data collection, and subsequently conducted the described three-part survey. The survey software DatStat Illume was used to electronically upload the data. No personal information that would identify the patient was collected via the electronic survey module.

### Study variables

**Fig. 1:**
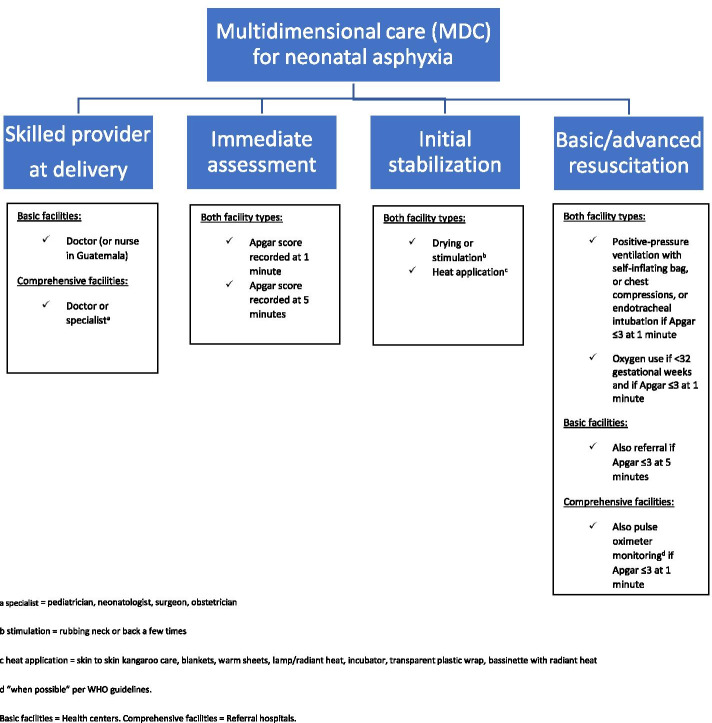
Definitions of SMI care practices for neonatal asphyxia

Multidimensional care (MDC) for neonatal asphyxia in health facilities, as measured in this analysis (Fig. 1), required four parts: 1) skilled provider present at delivery (physician, or nurse in Guatemala); 2) immediate assessment of the newborn (Apgar scoring at 1 and 5 min); 3) initial stabilization of the infant (drying or stimulation, heat application); 4) basic and advanced resuscitation if Apgar score ≤ 3 at 1 min (positive-pressure ventilation with self-inflating bag, or chest compressions, or endotracheal intubation, oxygen use if < 32 weeks’ gestational age). Additional resuscitation requirements were based on facility type (referral if delivery occurred at basic-level facility, pulse oximeter monitoring if delivery occurred at comprehensive-level facility). Multidimensional care for neonatal asphyxia was structured according to national norms from the ministries of health of each country and WHO international guidelines [1, 2, 11].

Covariates studied in this analysis were timing of data collection (baseline versus second-phase follow-up), country (Mexico as reference country, Nicaragua, Honduras, Guatemala), EmONC facility type (basic-level versus comprehensive-level), and disease severity (low birthweight, prematurity, and sepsis comorbidities versus asphyxia alone). Information on gestational age, maternal complications, and Apgar scores was obtained from the medical records.

### Statistical analysis

We conducted descriptive analysis and multivariable logistic regression analysis to examine potential patient-level, facility-level, and country-level factors associated with the primary outcome of multidimensional care coverage for neonatal asphyxia. Covariates included in the regression analysis were timing of data collection, country, EmONC facility type, and disease severity. We explored gestational age as a separate covariate but kept this integrated into the disease severity covariate. We also explored low Apgar score as a covariate but dropped this as it is part of the primary outcome. Regression models were adjusted for clustering of observations at the health facility level. No missing data were noted for the regression models. *P* values < 0.05 were considered significant. Stata 14.2 (StataCorp LP, College Station, TX, USA) was used for the analysis.

### Ethical considerations

This study received institutional review board approval by the University of Washington (exemption as non-human-subject research determination). The project was approved by data collection agencies (El Colegio de la Frontera Sur, Mexico), and by the ministries of health of participant countries (Guatemala, Honduras, Mexico, Nicaragua). Collaboration with the ministry of health, data-collection agencies, and indigenous communities within each country was maintained throughout the study. For the interview questionnaire and observation checklist sections of the survey, each facility health administrator participated in informed consent. The need for informed consent from patients for the study was waived by the University of Washington Institutional Review Board. For medical record extraction, patient information remained anonymized, and data were collected by trained professionals and uploaded electronically with DatStat Illume software.

## Results

The overall SMI design involved intervention and comparison groups. However, for this present analysis on asphyxia, not enough cases occurred in the comparison areas. As such, this analysis reflects intervention areas. Two hundred forty-eight neonates with asphyxia at time of delivery from 129 facilities were sampled for this analysis. Of these, 129 (52.0%) cases were collected at baseline and 119 (48.0%) cases at second-phase follow-up.

Table 1 depicts the medical record, patient, and facility characteristics of this population. While fewer comprehensive-level facilities (35 (27.1%)) existed, more asphyxia cases were managed in these facilities (155 (62.5%)). Facilities sampled per country ranged from 28 (21.7%) to 37 (28.7%), with the most cases obtained from Mexico (82 (33.1%)), followed by Nicaragua (74 (29.8%)), Honduras (49 (19.8%)), and Guatemala (43 (17.3%)), per facility quota. A minority of patients in this study were pre-term (< 37 weeks) gestational age (25 (11.5%)); with morbidities such as low birthweight, prematurity, and/or sepsis (56 (22.6%)); with maternal complications such as hemorrhage, pre-eclampsia, sepsis, and/or other condition (29 (18.2%)); or with low Apgar scores (≤3) at 1 min (66 (28.3%)) and 5 min (15 (6.4%)). Five neonatal deaths were recorded, with 4 (3.2%) of these cases at baseline and 1 (0.9%) case at second-phase follow-up. Availability of resuscitation equipment such as age-appropriate stethoscopes, self-inflating bags, intubating devices, and oxygen tanks ranged from 78 (75.0%) to 95 (84.1%) overall. While the composition of available staff remained relatively the same over time, with more nurses (122 (94.6%)) and general physicians (125 (96.9%)) than specialists (i.e., pediatricians, obstetricians, and anesthesiologists), relevant training in neonatal resuscitation and general newborn care increased from 49 (84.5%) to 70 (98.6%).

**Table 1:** Medical records, patient, and facility characteristics, by second phase follow-up

**Medical records characteristics**			
	**n (%)**		
	**Baseline** **129 (52.0)**	**Second phase** **119 (48.0)**	**Total**^a^ **248 (100)**
EmONC^b^ facility type			
Basic-level	20 (15.5)	73 (61.3)	93 (37.5)
Comprehensive-level	109 (84.5)	46 (38.7)	155 (62.5)
Country			
Mexico	47 (36.4)	35 (29.4)	82 (33.1)
Nicaragua	34 (26.4)	40 (33.6)	74 (29.8)
Honduras	21 (16.3)	28 (23.5)	49 (19.8)
Guatemala	27 (20.9)	16 (13.5)	43 (17.3)
**Patient characteristics**			
Gestational age			
< 37 weeks (pre-term)	15 (12.9)	10 (9.9)	25 (11.5)
> 37 weeks (full-term)	101 (87.1)	91 (90.1)	192 (88.5)
Comorbidities			
No recorded comorbidities	99 (76.7)	93 (78.2)	192 (77.4)
Low birthweight, prematurity, and/or sepsis	30 (23.3)	26 (21.9)	56 (22.6)
Maternal complications			
No recorded complications	52 (76.5)	78 (85.7)	130 (81.8)
Hemorrhage, pre-eclampsia, eclampsia, sepsis, and/or other	16 (23.5)	13 (14.3)	29 (18.2)
Apgar score at 1 min			
> 3	90 (74.4)	77 (68.8)	167 (71.7)
≤ 3	31 (25.6)	35 (31.3)	66 (28.3)
Apgar score at 5 min			
> 3	114 (94.2)	104 (92.9)	218 (93.6)
≤ 3	7 (5.8)	8 (7.1)	15 (6.4)
Neonatal outcome			
Alive	120 (96.8)	117 (99.2)	237 (97.9)
Dead	4 (3.2)	1 (0.9)	5 (2.1)
**Facility characteristics**			
	**n (%)**		
	**Baseline** 58 (45.0)	**Second phase** 71 (55.0)	**Total** 129 (100%)
EmONC facility type			
Basic-level	37 (63.8)	57 (80.3)	94 (72.9)
Comprehensive-level	21 (36.2)	14 (19.7)	35 (27.1)
Country			
Mexico	19 (32.8)	14 (19.7)	33 (25.6)
Nicaragua	8 (13.8)	23 (32.4)	31 (24.0)
Honduras	14 (24.1)	14 (19.7)	28 (21.7)
Guatemala	17 (29.3)	20 (28.2)	37 (28.7)
Resuscitation equipment			
Neonatal/pediatric stethoscope	33 (76.7)	62 (88.6)	95 (84.1)
Neonatal self-inflating bag	35 (83.3)	55 (78.6)	90 (80.4)
Neonatal laryngoscope, endotracheal tube	28 (82.4)	50 (71.4)	78 (75.0)
Oxygen tank	35 (83.3)	51 (72.9)	86 (76.8)
Personnel on staff			
Nurse	54 (93.1)	68 (95.8)	122 (94.6)
General physician	56 (96.6)	69 (97.2)	125 (96.9)
Pediatrician	25 (43.1)	32 (45.1)	57 (44.2)
Obstetrician	25 (43.9)	31 (43.7)	56 (43.8)
Anesthesiologist	21 (36.8)	28 (39.4)	49 (38.3)
Relevant training^c^	49 (84.5)	70 (98.6)	119 (92.3)

^a^ n may vary for each variable due to missingness

^b^ *EmONC* emergency obstetric and newborn care

^c^ Within last 3 years, training on neonatal resuscitation; general newborn care, temperature control, umbilical cord care; management of prematurity, low birth weight, sepsis, asphyxia; routine care of labor and delivery; basic care of obstetric emergencies

As shown in Table 2, after adjusting for covariates, odds of multidimensional care practices for neonatal asphyxia were significantly higher at second-phase follow-up compared to baseline (aOR = 2.4, 95% CI = 1.3–4.6). Additionally, Table 2 presents potential factors associated with the primary outcome adjusted by round, country, facility level, and disease severity in a multivariable model. Odds of multidimensional care compliance for neonatal asphyxia were significantly higher in Honduras (aOR = 4.0, 95% CI = 1.4–12.0) as compared to Mexico. Facility- and patient-level covariates were found to have a positive, but not significant, correlation to the primary outcome.

**Table 2:** Adjusted odds ratios for receiving multidimensional care (MDC) for neonatal asphyxia by measurement, country, facility level, and disease severity

	Adjusted OR^a^ (*n* = 248)	(95% CI)
Measurement		
Second-phase	**2.4**	**(1.3–4.6)**
Baseline	*ref*	
Country		
Nicaragua	1.4	(0.7–2.9)
Honduras	**4.0**	**(1.4–12.0)**
Guatemala	0.8	(0.4–1.8)
Mexico	*ref*	
Facility level		
Comprehensive facilities	1.8	(0.8–3.8)
Basic facilities	*ref*	
Disease severity		
Comorbidities (low birthweight, prematurity, sepsis)	1.0	(0.2–1.2)
Asphyxia only	*ref*	

Basic facilities = Health centers. Comprehensive facilities = Referral hospitals

^a^ variables controlled for included timing of data collection, country, facility type, and disease severity

In Table 3, we provide descriptive statistics of multidimensional care coverage for neonatal asphyxia over time. Implementing multidimensional care for newborn respiratory failure in SMI areas rose from 51.9% at baseline to 68.1% by second-phase follow-up, increasing coverage by 16.2 percentage points. All countries improved multidimensional care coverage of neonatal asphyxia over time, ranging from 50.0% of cases in Guatemala to 85.7% of cases in Honduras by second-phase follow-up. Mexico showed the most progress, by 21.0 percentage points from baseline (44.7%) to second-phase follow-up (65.7%). While both facility types improved multidimensional coverage of neonatal asphyxia over time, starting at a similar baseline coverage, more advancement (23.8 percentage points) was seen in comprehensive-level facility cases (76.1%) by second-phase follow-up. Likewise, multidimensional care coverage of neonatal asphyxia increased over time across all patients. Notably, greater improvement (33.1 percentage points) was observed in higher-risk patients (associated low birthweight, prematurity, or sepsis comorbidities), with multidimensional care levels reaching 73.1% by second-phase follow-up.

**Table 3:** Multidimensional care (MDC) for neonatal asphyxia, by second-phase followup

	% (95% CI)	
	Baseline	Second phase
Multidimensional care (MDC) for neonatal asphyxia	51.9 (43.0–60.8)	68.1 (58.9–76.3)
Country		
Mexico	44.7 (30.2–59.0)	65.7 (47.8–80.9)
Nicaragua	52.9 (35.1–70.2)	65.0 (48.3–79.4)
Honduras	76.2 (52.8–91.8)	85.7 (67.3–96.0)
Guatemala	44.4 (25.5–64.7)	50.0 (24.7–75.4)
Facility level^a^		
Basic facilities	50.0 (27.2–72.8)	63.0 (50.9–74.0)
Comprehensive facilities	52.3 (42.5–62.0)	76.1 (61.2–87.4)
Disease severity		
Asphyxia only	55.6 (45.2–65.6)	66.7 (56.1–76.1)
Comorbidities (low birthweight, prematurity, and/or sepsis)	40.0 (22.7–59.4)	73.1 (52.2–88.4)
Multidimensional care (MDC) components^b^		
Skilled provider at birth	79.1 (71.0–85.7)	92.4 (86.1–96.5)
Immediate assessment	93.8 (88.2–97.3)	93.3 (87.2–97.1)
Initial stabilization	77.5 (69.3–84.4)	79.0 (70.6–95.9)
Basic/advanced resuscitation	38.7 (21.8–57.8)	65.7 (47.8–80.9)
Basic/advanced resuscitation requirements^c^		
Referral in basic facilities	100.0 (47.8–100)	76.2 (52.8–91.8)
PPV ETT CPR	71.0 (52.0–85.8)	91.4 (76.9–98.2)
Oxygen use (if < 32 weeks gestational age)	NULL	50.0 (1.3–98.7)
Pulse oximeter use in comprehensive facilities	34.6 (17.2–55.7)	78.6 (49.2–95.3)

^a^ Basic facilities = Health centers. Comprehensive facilities = Referral hospitals

^b^ Skilled provider at birth = doctor (or nurse in Guatemala) in basic facilities, or specialist (pediatrician, neonatologist, surgeon, obstetrician) in comprehensive facilities. Immediate assessment = Apgar score recorded at 1 min and 5 min. Initial stabilization = drying or stimulation; heat application. Basic/advance resuscitation if Apgar score ≤ 3 at 1 min = ventilation with self-inflating bag, or chest compressions, or endotracheal intubation

^c^ Referral if Apgar score ≤ 3 at 5 min if at basic facility. *PPV* positive-pressure ventilation, *ETT* endotracheal tube, *CPR* cardiopulmonary resuscitation

Table 3 also illustrates clinical performance, by breakdown of the multidimensional care components. While skilled provider at delivery, immediate assessment, and initial stabilization components reached or remained at high levels by second-phase follow-up, the resuscitation component achieved the most progress, increasing from 38.7% use at baseline to 65.7% coverage levels by second-phase follow-up. Further breakdown of the resuscitation component shows the main criteria of positive-pressure ventilation, chest compressions, or endotracheal intubation that applied to all low Apgar patients reached levels of 91.4% by second-phase follow-up. Other resuscitation components, relevant to a subset of patients, such as oxygen administration and pulse oximeter use increased to 50.0 and 78.6%, respectively, over time.

## Discussion

SMI significantly improved equitable coverage and quality care for newborns in the most impoverished areas of Mesoamerica and should serve as a model elsewhere. In terms of coverage, multidimensional asphyxia care improved across all countries, facility types, and patient risk stratification. In terms of quality of care, the resuscitation component advanced the most following SMI interventions, acting as a key factor in achieving comprehensive asphyxia care. SMI drivers for such outcomes and consideration of a sustainable model for similar environments may be attributed to a country-led performance-based approach, health system strengthening using a stepwise process, and training personnel within broader quality improvement strategies.

SMI asphyxia care coverage can be adapted to various clinical contexts among low-income populations. A significant increase of multidimensional care coverage in the country with a larger proportion of neonatal mortality for under-5 children [3], and greater progress in higher-acuity facilities and sicker patients was shown in this analysis. Using a country-led performance-based approach, participating ministries of health took ownership and accountability [12] by prioritizing multidimensional asphyxia care, setting coverage target goals, formalizing normative changes, and allocating budgets. SMI is also a regional endeavor, with healthy competition [12] from other countries as a motivating factor. These findings offer potential to scale up to like environments in Mesoamerica, and regions such as sub-Saharan Africa and Asia with higher neonatal mortality burden [4].

SMI demonstrates that more complex asphyxia care is attainable and should be expected, in order to raise the standard of care for vulnerable groups worldwide. SMI used a stepwise strengthening approach to achieve more complex care by strategically investing in limited resources. In the first phase of SMI, our analysis noted an increase in health system inputs, with sustained staffing of non-specialists and, to a lesser degree, availability of resuscitation equipment. In the second phase, results showed an increase in training of providers in neonatal resuscitation and routine delivery care, among other interventions. The more complex component of asphyxia care, that is resuscitation, was possible in part due to ensuring existing skilled providers were mobilized to bedside, along with maintaining high levels of timely assessment and stabilization. In combination, these SMI factors optimally utilized limited resources, developed competency and capacity building, and created a supportive infrastructure to improve the resuscitation component, and thereby multidimensional care for asphyxia collectively, applicable to any resource-constrained setting.

In addition to a country-led approach and a stepwise process for health system strengthening, SMI gains may be attributed to its real-life setting of measuring and evaluating its multidimensional asphyxia care intervention. For example, SMI quality improvement strategies offered higher-level solutions such as certifying general physicians and nurses on asphyxia care to address workforce shortages, and creating flowcharts that designated timely workflow steps, personnel roles, resuscitation equipment in delivery rooms, and high-risk delivery alerts. Other studies, most notably the Helping Babies Breathe (HBB) program implemented worldwide with positive outcomes [13, 14], often focused on simulation-based training without ongoing assessment of the broader environmental factors of health facilities. Incorporating real-life context beyond training has been limited [13, 15, 16]. Of the initiatives with real-life audits, only execution of clinical care was examined, and not broader-based quality improvement cycles that may aid in improving decision-making, teamwork, and process-oriented needs. Additionally, multidimensional components such as advanced resuscitation or skilled provider at bedside were less emphasized in these studies [17–21]. Thus, this SMI analysis characterizes the performance gap of real-time newborn asphyxia care using ongoing quality improvement strategies, rather than simulated training alone, for the most impoverished within countries and regionally.

We acknowledge limitations to this study. First, the SMI evaluation design planned for intervention and comparison groups. However, for this present study on asphyxia, insufficient cases were available in the comparison group for all the countries, which led to the decision to restrict analysis to intervention areas. General conditions of access and service provision have not changed overall in recent years in these countries, suggesting the changes observed may be attributable to SMI. Second, due to a separate survey module, healthy neonates were not included in this analysis. We cannot assess the proportion of neonates with asphyxia among all newborns delivered in low-income areas. Third, in evaluating quality of care, we strived to capture multidimensional care, but by no means complete care. For example, not all components of resuscitation were measured in the survey, such as adequacy of ventilation by chest rise or the administration of life-saving medications. Yet, SMI captures most aspects of international standards of resuscitation and reflects the performance indicators agreed upon by participating countries.

## Conclusion

Overall, SMI offers a country-led performance-based approach, a health system strengthening process, and training within broader quality improvement strategies to address the clinical performance gap of neonatal care in resource-limited hospitals in Mesoamerica. SMI is a promising model that should be applied to high neonatal mortality regions, due to its adaptability to various clinical settings and ability to raise the standard of care. Programs such as SMI are needed to promote timely, effective, and equitable care of newborns during the intrapartum period worldwide.

## Data Availability

Availability of most data for Salud Mesoamérica Initiative baseline and follow-up surveys used in this study are publicly available at the Global Health Data Exchange (GHDx) website: http://ghdx.healthdata.org/. Any survey data that is not yet public can be requested from the IHME research team. All data will be made public by the conclusion of the Initiative.

## Abbreviations

EmONC: Emergency Obstetric and Newborn Care
HBB: Helping Babies Breathe
ICD-10: International Classification of Disease coding
MDC: Multidimensional care
SMI: Salud Mesoamérica Initiative
